# Spatio-temporal cluster analysis of the incidence of *Campylobacter *cases and patients with general diarrhea in a Danish county, 1995–2004

**DOI:** 10.1186/1476-072X-8-11

**Published:** 2009-02-20

**Authors:** Martin Rudbeck Jepsen, Jacob Simonsen, Steen Ethelberg

**Affiliations:** 1 Department of policy analysis, National Environmental Research Institute, University of Aarhus, Roskilde, Denmark; 2 Department of Epidemiology, Statens Serum Institut, Copenhagen, Denmark; 3 Department of Epidemiology Research, Statens Serum Institut, Copenhagen, Denmark; 4 Department of Bacteriology, Mycology and Parasitology, Statens Serum Institut, Copenhagen, Denmark

## Abstract

*Campylobacter *infections are the main cause of bacterial gastroenteritis in Denmark. While primarily foodborne, *Campylobacter *infections are also to some degree acquired through other sources which may include contact with animals or the environment, locally contaminated drinking water and more. We analyzed *Campylobacter *cases for clustering in space and time for the large Danish island of Funen in the period 1995–2003, under the assumption that infections caused by 'environmental' factors may show persistent clustering while foodborne infections will occur randomly in space. Input data were geo-coded datasets of the addresses of laboratory-confirmed *Campylobacter *cases and of the background population of Funen County. The dataset had a spatial extent of 4.900 km^2^. Data were aggregated into units of analysis (so-called features) of 5 km by 5 km times 1 year, and the *Campylobacter *incidence calculated. We used a modified form of local Moran's I to test if features with similar incidence rates occurred next to each other in space and time, and compared the observed clusters with simulated clusters. Because clusters may be caused by a high tendency among local GPs to submit stool samples, we also analyzed a dataset of all submitted stool samples for comparison. The results showed a significant persisting clustering of *Campylobacter *incidence rates in the Western part of Funen. Results were visualized using the Netlogo software. The underlying causes of the observed clustering are not known and will require further examination, but may be partially explained by an increased rate of stool samples submissions by physicians in the area. We hope, by this approach, to have developed a tool which will allow for analyses of geographical clusters which may in turn form a basis for further epidemiological examinations to cast light on the sources of infection.

## Background

*Campylobacter *is the leading cause of bacterial gastroenteritis in Europe, North America and Australia and the annual number of infections reported in these continents has generally been increasing during the past 2 to 3 decades [1-3]. In Denmark the incidence of *Campylobacter *infections rose 4-fold from 1991 to 2001, but has since stabilized; in 2001 there were 87 registered infections per 100,000 population; the 2007 incidence was 71.5 per 100,000 population [4].

The major part of the infections are sporadic and consumption and handling of fresh poultry, chicken in particular, is generally recognized as the most important source of infection [2,3]. A recent Danish case-control study of sporadic infections found consumption of fresh (as opposed to frozen) chicken to be the most important risk factor along with foreign travel [5]. However, the epidemiology of *Campylobacter *is not thoroughly understood and a number of other sources are believed to play a role, including: drinking water, the environment and contact with farm animals. In Denmark, as in many other countries, such sources of infection would be expected to be geographically unevenly distributed, i.e. localized to certain areas, with the source of infection potentially persisting through time. This is in contrast to a situation where consumption of poultry is the only source, since the risk of buying contaminated poultry is the same throughout the country. Epidemiological studies examining the spatial distribution of human *Campylobacter *patients may therefore be valuable both as general tools to cast light on non-food risk factors and to search for specific areas where there might be an increased risk of infection. Though 'environmental' risk factors (such as contact with farm animals) have been identified in a number of case-control studies [3], only few studies using spatial analysis have been performed. In a recent Canadian study of the location of *Campylobacter *patients in the province of Manitoba, patient and population data were aggregated on health municipalities and SaTScan analysis used to find clusters of increased incidence while an association with farm animals was found using regression analysis; indicating that different routes of infection were at play in different parts of the province [6].

In a Danish setting, a previous study have shown an increased risk of infection in rural as opposed to urban areas, in particular among children [7] and in another study, examining individual addresses of *Campylobacter *patients, an above normal risk of episodes of infection occurring on some addresses was found, suggesting the possible presence of environmental risk factors at these addresses [8].

In this study our objective was to perform a geographical and temporal cluster analysis of the incidence of human *Campylobacter *infections using a local indicator of spatial association [9]. Our aim was to pinpoint areas with an increased risk of infection in order to assist epidemiologists in finding specific sources of infection. We also aimed to develop a methodologically transparent analytical tool while simultaneously considering and discussing the number of potential biases which might affect our analysis.

## Methods

### Data material

**Figure 1:**
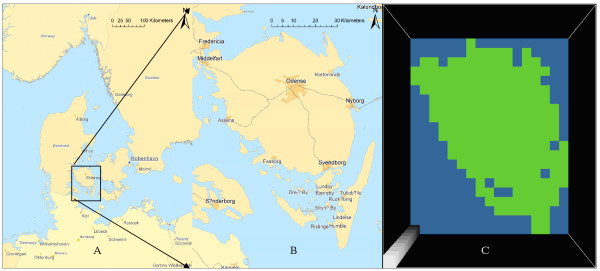
**Geographical map of (A) Denmark, (B) the study area, the Danish island of Funen and its surrounding smaller islands and (C) the study area divided into 5 by 5 km cells**.

The study period was from 1 January 1995 to 31 December 2003. During this period Denmark was divided into 16 counties, each of which was generally served by only one clinical microbiological laboratory. In order to avoid a possible laboratory bias we choose to restrict our study to only one county and thereby one laboratory. We choose the county of Funen which has natural geographic borders; it consists of one main island in addition to several smaller islands (Figure 1). The population of Funen County ranged from 468,099 in 1995 to 473,852 in 2003 [10]. During the study period Funen County was served by the clinical microbiological laboratory at Statens Serum Institut. All fecal samples submitted for analysis for gastrointestinal pathogens – both from GPs and hospitals – from Funen County were referred to this laboratory.

Data from the Statens Serum Institut laboratory systems were extracted as described elsewhere [11]. The samples were identified and geocoded to exact address using the CPR (Central Person Registration) number, which is a ten digit unique identifier given to all individuals with permanent residency in Denmark [12]. Two datasets were established. One consisted of all individuals who had had one or more stool samples submitted for microbiological analysis, from a physician from Funen County regardless of the result of the laboratory analysis (n = 50,049), in the following referred to as diarrhea cases. The second dataset consisted of all individuals with a positive *Campylobacter *diagnosis, henceforth referred to as *Campylobacter *cases (n = 2,984). If more than one sample (diarrhea cases) or positive sample (*Campylobacter *cases) existed for the same individual, only the first within a 12 month period was retained. In addition to *Campylobacter *and diarrhea cases, the background population of Funen County was similarly geocoded to exact address. We searched for clustering of the *Campylobacter *cases and subsequently performed an identical cluster detection on the diarrhea cases to visually check if clustering co-occurred spatio-temporally for the two datasets, because this could point to a potential sampling bias when searching for *Campylobacter *clusters. We return to this problem in the discussion section.

The dataset had a spatial extent of 4,900 km^2 ^and we aggregated all data in a grid with a resolution of 5 by 5 km per cell resulting in 209 cells of 25 km^2 ^each. We arrived pragmatically at this spatial resolution. Using 1 by 1 km cells resulted in a large number of cells neither containing any population nor any *Campylobacter *cases, while 10 by 10 km cells were too large, evening out local variations in *Campylobacter *incidences and thereby hiding potential point sources in small, local areas. While we are aware of the modifiable area unit problem described by Openshaw [13], we have not quantitatively analyzed the impact of changing size and shape of the cells (the units of analysis) on our results and acknowledge this as a potential shortcoming of this study.

**Figure 2:**
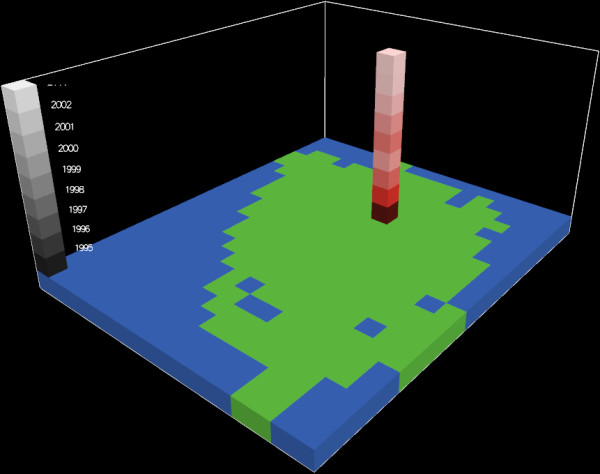
**A nine year stack of data highlighting the province capital Odense**.

Combining the 25 km^2 ^cells with a one year temporal resolution of the nine year period covered (1995–2003) resulted in a three-dimensional data frame, where the unit of analysis is a space-time cell with the dimensions 25 km^2 ^* 1 year, henceforth referred to as a *feature*. In total, the data frame was composed of 1,881 features (209 * 9). The space-time continuum was thus divided into discrete units where the third spatial dimension, elevation, was substituted for time. In the following, we will refer to all features within a given year as a 'layer' and to all features from the same geographical location as a 'stack'. Figure 2 shows the data frame with the study area (corresponding to a layer) demarked in green and a nine year 'stack' of data for the 25 km^2 ^area covering Odense, the major city on Funen.

### Data Analyses

Spatial- and spatio-temporal cluster analyses can be performed in various software applications, of which SaTScan (the Spatial and Space-Time Scan Statistics)[14] is probably the most widely used within public health and ArcGIS from ESRI is the dominant commercial application. Analyzing the distribution of disease incidences in time and space is associated with a large number of methodological challenges, of which we list a few here: First, in order to calculate incidences, a population denominator must be specified. As described above, we used a unit of analysis of 5 by 5 km times 1 year for aggregation of cases and population respectively, but there are large analytical impacts of deciding on how to combine spatial and temporal dimensions. Second, when calculating *Campylobacter *incidences, features with few cases and a small population will result in disproportionally high incidences [15-17]. Third, defining spatial associations between the phenomena under study is not straightforward and clusters could be searched for in cubic, cylindrical, conical or other shaped windows [18] with various levels of distance decay.

Having no hands-on experience with SaTScan we decided to construct our own tool using the software Netlogo [19] for the present purpose. This approach had the advantages that we had *a priori *experience with Netlogo, which has build-in 3D data visualization options, that we could control the data flow through the model and that we had perfect knowledge of all data processing steps.

We assumed that foodborne *Campylobacter *infections are independent events occurring randomly in space and time while *Campylobacter *cases associated with an environmental factor are grounded in space expressing spatio-temporal autocorrelation. This phenomenon has been put forward by Tobler [20] as "*Everything is related to everything else, but near things are more related than distant things*" and is sometimes referred to as Tobler's Law, or the first law of Geography. To detect campylobacteriosis associated with other factors than food we therefore searched for features where the incidence of *Campylobacter *infections deviated from the mean incidence, and where the incidence of the neighboring features (in time and space) also deviated from the mean incidence. To do so, we used the algorithm for computing local spatial autocorrelation developed by Luc Anselin [9,21,22]:

(1) $$I_{i}=\frac{\left(x_{i}-\bar{x}\right)}{\sum_{i}{\left(x_{i}-\bar{x}\right)}^{2}}\cdot \sum_{j}w_{ij}\left(x_{j}-\bar{x}\right)$$

Where *I*_*i *_is the local Moran's Index computed for each feature *i*, *x*_*i *_is the incidence for feature *i*, $\bar{x}$ is the mean incidence for the entire dataset, *x*_*j *_is the incidence for the neighbor and *w*_*ij *_is a weighting function for all pairs of *i *and *j*.

*I*_*i *_can be either positive or negative. A) If the incidence in feature *i *(the target feature) is higher than the mean incidence, and the mean incidence of the neighboring features is also above the mean incidence, *I*_*i *_will be positive. B) If both the incidence of the target feature and the mean incidence of the neighbors are below the mean incidence, *I*_*i *_will also be positive, because it is the product of the negative deviations from the mean. In both cases, A and B, there is a spatial cluster of similar values and positive spatial autocorrelation. C) If the incidence of the target feature is higher than the mean incidence and the mean incidence of the neighboring features is lower than the mean incidence, or D), vice versa, *I*_*i *_will be negative indicating spatial dissimilarity, or negative spatial autocorrelation. Situations A and B are sometimes referred to as high/high and low/low clusters, while C and D are called high/low and low/high, respectively.

**Figure 3:**
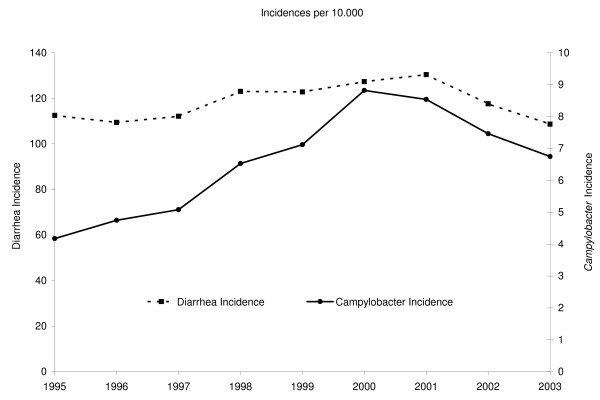
**Incidences per 10,000 of diarrhea (dotted line) and *Campylobacter *(solid line)**.

In the study period there was a secular trend that, if not corrected for, in itself would affect the analyses. The input data for the analyses shows two similar "inverted U"-shaped trends with initial increases in the number of *Campylobacter *cases and also, although to a lesser extend, in the number of diarrhea cases followed by a decrease in numbers toward the end of the study period (Figure 3). Uncorrected, this temporal trend could lead to identification of clusters in the middle of the study period when the incidences peak. As we considered this peak a global phenomenon within the study area we therefore de-trended the data during the analysis by replacing $\bar{x}$ of Equation 1 with the mean incidence for a given year (${\overset{⌢}{x}}_{it}$ and ${\overset{⌢}{x}}_{jt}$) (Equation 2).

In our analyses we expanded the neighbor weighting function, *w*_*ij*_, to take spatial as well as temporal neighbors into account. We used a cubic Moore neighborhood definition of 26 neighbors but restrained the neighborhood to only include features with *Campylobacter *cases. The implications on the analyses of excluding zero-activity features are 1) we avoid detection of zero-activity (low/low) clusters. If not excluded, a target feature and its neighbors which don't have any *Campylobacter *cases will yield negative values for *Z*_*it *_and *Z*_*jt *_(Equation 3) and result in a positive *I*_*it *_value indicating a cluster. 2) We also reduce detection of spatial dissimilarity (low/high and high/low clusters) to only detect dissimilarities between *Campylobacter *positive features.

In features with a small population, a single *Campylobacter *case will result in a disproportionally high incidence which would likely bias the analysis. To correct for this potential bias, we replaced s^2 ^for each feature with a variance estimate that took the feature's population into account and calculated the deviation of the feature's incidence from the mean incidence of the given year, divided by the square root of the variance estimate:

(2) $$Z_{it}=\frac{x_{it}-{\hat{x}}_{it}}{\sqrt{{\left(\frac{1}{p_{it}}-\frac{1}{p_{\cdot t}}\right)}^{2}\cdot p_{it}\cdot \left({\hat{x}}_{it}\right)+{\left(\frac{1}{p_{\cdot t}}\right)}^{2}\cdot \left(p_{\cdot t}-p_{it}\right)\cdot {\hat{x}}_{it}}}$$

where *p*_*it *_is the population of the *i*th feature at time *t*, *p*_·*t *_is the sum of the population of features at time *t *and ${\overset{⌢}{x}}_{it}$ is the expected incidence for the *i*th feature at time *t*, calculated as the mean incidence for time *t*.

The resulting equation for calculating local Moran's I for a given feature to a given time thus became:

(3) $$I_{it}=Z_{it}\cdot \sum_{j}w_{ij}\left(Z_{jt}\right)$$

To estimate a statistical significance of *I*_*it *_we calculated the expected *I*_*i*_, *E(I*_*it*_) given no spatial autocorrelation:

(4) $$E\left(I_{it}\right)=\frac{-\sum_{j}w_{ij}}{\left(n-1\right)}$$

where *n *is the count of features in the data frame. This was subtracted from the calculated *I*_*it*_, and divided by the standard deviation of the features:

(5) $$Z\left(I_{it}\right)=\frac{I_{it}-E(I_{it})}{\sqrt{\mathrm{var}\left(I_{it}\right)}}$$

Throughout the analyses we used a Z-score of 2.76 (99% confidence level) and to further sort out 'misclassified' features identified as significant clusters, we passed the features through two logical tests: 1) to avoid cluster features which could be caused by transient outbreaks, cluster features should have at least one other cluster feature in its stack and, 2) to avoid 'coldspot' clusters, the incidence of the cluster feature should be larger than the mean incidence of the 3 by 3 stack surrounding the feature. Finally, the strength of our approach was tested in a permutation analysis: Using a Poisson distribution, we generated 10,000 permutations of the spatio-temporal distribution of cases in the study area based on the population of each feature, and restrained the expected number of simulated cases to match the total number of observed cases. We recorded the number of accepted cluster features for each permutation, and used the simulated data to test if the observed number of clusters differed significantly from what could occur by random.

Data analyses and visualizations were performed in *Netlogo*, a software capable of performing 3D visualization [19]. While originally being developed for the programming of agent-based models, the cellular automata facilities of this software also allow for cell operations such as calculations on spatial relations.

## Results

Table 1 shows the normalized incidences for the diarrhea and *Campylobacter *cases for the period 1995–2003, with the year 1995 as reference, while Figure 3 shows the incidence of diarrhea and *Campylobacter *per 10,000 population for the same period. The diarrhea incidence shows a slight increase and subsequent decrease during the study period, with an initial value in 1995 of 5255 cases and a peak in 2001 of 6140 cases. The *Campylobacter *incidence shows a similar, albeit more drastic trend with more than a doubling of the incidence from 1995 (195 cases) to 2000 (415 cases).

**Table 1:** Normalized incidences (1995 = 1.00) for the two input data sets shown in Figure 6.

	1995	1996	1997	1998	1999	2000	2001	2002	2003
DiarrheaIndex	1.00	0.97	1.00	1.09	1.09	1.13	1.16	1.05	0.97
CampyIndex	1.00	1.14	1.22	1.56	1.71	2.11	2.04	1.79	1.62

**Figure 4:**
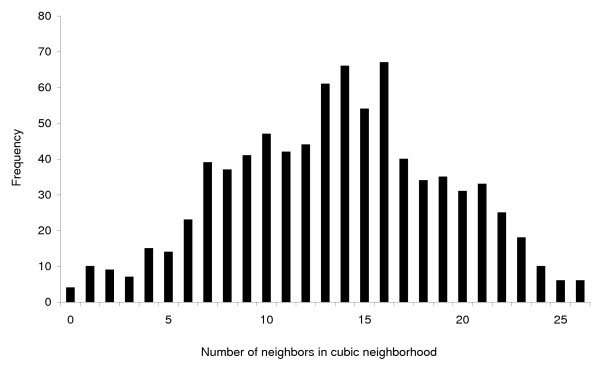
**Distribution of neighborhood sizes for the 818 features with one or more *Campylobacter *cases**. The x-axis shows the number of neighboring features (j) per target feature (i) and the y-axis shows how often a given neighborhood size is observed.

**Figure 5:**
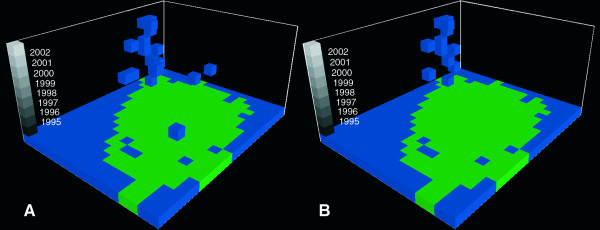
**(A) Spatio-temporal distribution of features identified as *Campylobacter *clusters using Equation 3 with a Z-score of 2.76**. (B) Distribution of *Campylobacter *cluster features passing the tests for outbreaks and cold spots.

**Figure 6:**
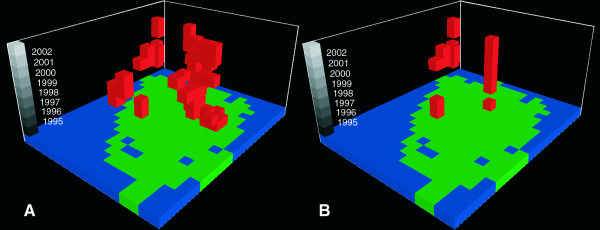
**(A) Spatio-temporal distribution of features identified as diarrhea clusters using Equation 3 with a Z-score of 2.76**. (B) Distribution of diarrhea cluster features passing the tests for outbreaks and cold spots.

**Figure 7:**
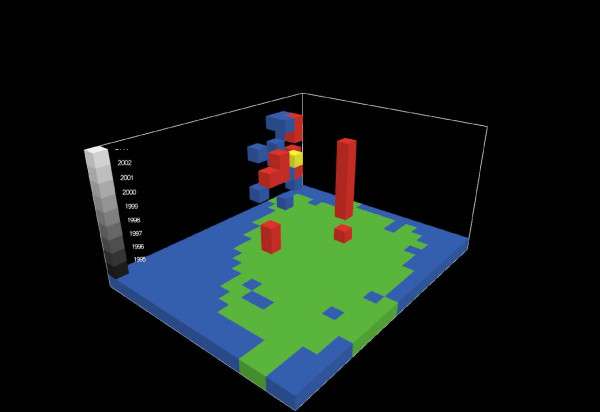
**Spatio-temporal distribution of features with significant clustering**. Blue = *Campylobacter *cluster, red = diarrhea cluster and yellow = both.

Of the 1,881 features in the data set, 818 (44%) contained at least one *Campylobacter *case and only four of the 818 did not have any other features containing *Campylobacter *cases in their neighborhood (Figure 4). The mean number of *Campylobacter *positive features in the neighborhood was 13.5. Applying the modified local Moran's I (Equation 3) to the *Campylobacter *dataset resulted in 19 features being identified as clusters (Table 2). Passing these *Campylobacter *clusters through the logical tests reduced the result list to 12 features; two cluster features are located in the central Western part of the study area while the remaining ten forms a larger aggregation of cluster cells in the North-Western part of the study area. The cluster aggregation spans four adjacent stacks or a spatial extend of 100 km^2^, and covers the period 1996; 1998–2003 (Figure 5). We performed the same analysis on the diarrhea cases resulting in 57 features before the logical tests and 18 features after passing the clusters through the logical tests. Eight of these are located in the same stack, covering the central part of Odense, the provincial capital, for the period 1995; 1997–2002. Four of the diarrhea cluster features are placed South-West of the center of the study area and the remaining six are found near or in the *Campylobacter *cluster aggregation in the North-Western part of the study area (Figure 6). Of these six diarrhea cluster features one overlaps spatio-temporally with the *Campylobacter *cluster features (Figure 7).

**Table 2:** Features identified as significant *campylobacter *clusters using Equation 3 and logical tests.

x	y	z	Population	*Campylobacter *cases	Diarrhea cases	*Campylobacter *cluster	Diarrhea cluster
3	13	6	623	3	9	X	
3	13	7	627	2	3	X	
3	13	9	629	3	12	X	
3	14	2	639	2	7	X	
3	14	8	618	2	12	X	
3	14	9	615	2	13	X	
3	15	4	2461	7	56		X
3	15	6	2474	5	50		X
4	13	5	673	2	13	X	
4	13	9	686	3	11	X	
4	14	4	2259	4	19	X	
4	14	5	2294	3	44		X
4	14	6	2283	6	52	X	X
4	14	8	2279	2	39		X
4	14	9	2307	4	41		X
5	9	5	130	1	2	X	
5	9	8	128	1	2	X	

The table also provides information on Diarrhea cluster features located close to, or overlapping with, the *Campylobacter *feature clusters.

**Figure 8:**
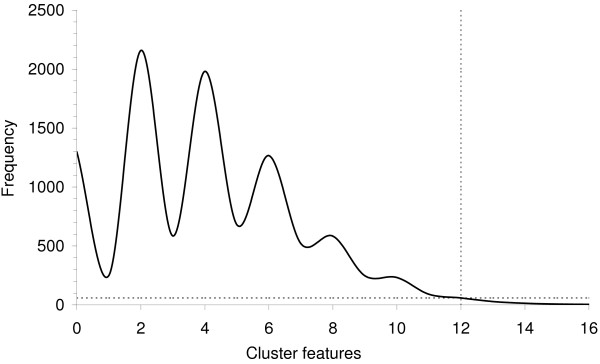
**Frequencies of cluster sizes for 10,000 simulated *Campylobacter *distributions**. The x-axis shows the number of features comprising a cluster (cluster size) and the y-axis the number of permutations yielding the cluster size indicated on the x-axis. Dotted lines indicate the number of cluster features (n = 12) found in the observed *Campylobacter *data.

For the permutation analysis the observed number of *Campylobacter *cases was spatio-temporally allocated in the features according to the population of each feature using a Poisson distribution, tested for clustering using Equation 3 and filtered through the logical tests. Slightly more than one percent (n = 114) of the 10,000 permutations resulted in more than the 12 *Campylobacter *cluster features we observed in the *Campylobacter *dataset (Figure 8). Therefore the result of this analysis indicated that the clustering of *Campylobacter *features is statistically significant.

## Discussion

We have used Netlogo to construct a tool for calculation and visualization of clusters of disease incidence. Visualizing the data has proven very useful because it provides an insight into the spatio-temporal distribution of data that might otherwise have gone unnoticed (see additional file 1). Compared with cluster detection functionability found in professional software applications such as for instance ArcGIS and SaTScan, the relatively transparent coding of the functions in our tool have provided us with a thorough control of the data processing and allowed for flexible customization of the equations used for detecting clusters. The local Moran's I is a widely used index for identifying local spatial clusters. It identifies features for which the incidence is similar to the incidence in adjacent features and where the incidence deviates from the global incidence. We extended the index to search for temporal clusters as well in order to identify spatio-temporal aggregations of *Campylobacter *cases in the study area. We also replaced the traditional variance estimate (Equation 1) with the modified variance estimate (Equation 3) in order to account for heterogeneous population sizes among the features and to avoid very high incidence rates in features with low background populations, and were able to identify a large aggregation of *Campylobacter *cluster features which could not be reproduced by simulating randomly distributed cases. When analyzing diarrhea cases identically, we found a partial spatio-temporal overlap between *Campylobacter *and diarrhea cluster features.

An underlying premise in this study has been that foodborne *Campylobacter *infections are transient point phenomena randomly distributed in time and space, while infections related to other factors may exhibit a persisting pattern in time and space. Therefore, a detection of specific geographical clusters of infection is valuable as it may lead to identification of specific risk factors in a given area or cast light on the importance of different sources of infection. We identified one part of the island of Funen in particular, where clustering occurred (Figure 5). This area, located in the North-Western part of the island, is predominantly rural. It is possible that a consistent surplus of cases of *Campylobacter *in a given area is due to environmental factors, for instance related to the presence of farms in the area or problems with the water supply. The findings in this study could serve as a starting point for further epidemiological studies in our study area. A first step could be to contact local general practitioners and regional environmental authorities in order to uncover the causes for these findings. One possibility which we were able to address through our analysis, however, was that the cluster of increased *Campylobacter *incidence might coincide with clustering of general diarrhea incidence. In fact we found this to be partly the case. Such clustering may be due to persistently increased occurrence of diarrheal disease in the area, again indicating a potential environmental problem, or, we speculate, may be due to a special interest among general practitioners in uncovering the etiology of diarrhea in their district. In either case, it's possible that a persistent high diarrheal sampling frequency have contributed in producing the observed *Campylobacter *cluster. This is because examination of more stool samples is likely to lead to the diagnosis of more positive cases.

Like other epidemiological analyses, geographical epidemiological analyses are subject to a series of potential biases. In planning this study we noted several potential pitfalls that might distort our analyses. These related to: underlying population structure, differences in laboratories' testing, secular trends, point source outbreaks, differences in sampling frequency among physicians and finally the possibility that patients become infected other places than at their residential addresses. Below, we describe how we took each of these potential problems into account in our analyses.

1) Detected clusters of disease-cases might simply reflect areas with high population density, such as major cities. Therefore we needed to take the underlying population structure into account. Because the address of all Danish residents are registered in the central population register and these addresses have been geo-coded, we where able to work with incidence of disease by summarizing cases and the underlying population in cells in a defined grid net.

2) Different clinical microbiology laboratories may use different methodologies and standards and also reporting differences may exist. If a given area is served by several laboratories and some of these use more sensitive methodologies than others, this will be reflected in an uneven distribution of cases. In Denmark, such differences are believed to be minor, but historically some reporting differences may have occurred. Therefore, in this study we confined our analysis to an area served by one laboratory only.

3) Long-term changes in the incidence of the disease under study might introduce a bias. In our study there was a clear temporal trend as the number of cases changed during the study period (Figure 3). We took care to account for this in the cluster calculations.

4) Outbreaks caused by the infectious disease under study might introduce a separate level of complication in the analyses. The purpose of a geographical analysis may well be to detect outbreaks, but the purpose may also – as in the present study – be to try to identify geographic areas with an inherent increase in risk of infection. In such an instance, outbreaks need to be adjusted for, as their presence may be confused for clusters caused by the local geographical factors. In the case of *Campylobacter*, this problem is not so significant because *Campylobacter *only very rarely cause outbreaks As far as we are aware, there were no registered general outbreaks involving laboratory confirmed *Campylobacter *cases in Funen County during the study period [23,24]. Nevertheless, unrecognized outbreaks may have occurred and it was therefore necessary to take these into account. We did this by distinguishing between transient and persistent clusters; transient clusters were assumed to be outbreaks caused by factors (most likely food) not associated with the place they occurred as such, and removed from the analyses.

5) A bias may arise because the majority of gastro-intestinal disease episodes that occur are not captured for analysis as they are not registered. In order for an episode of campylobacteriosis to be registered, an individual needs to be infected, develop symptoms, be seen by a physician, be judged by the treating physician as requiring a stool sample analysis, and, furthermore, the laboratory shall successfully grow the organism and report the finding. The patients under study are thus a selected sample of all patients and factors influencing the selection process may lead to 'artificial' geographical clustering of cases. In a recent inventory of the diagnostic sensitivity of the Statens Serum Institut laboratory 3.2% on average of submitted samples were found positive for *Campylobacter *[11]; in our study there were 17 times more submitted samples than *Campylobacter *positives. Because we had direct access to the laboratory database-systems, we believe that the reporting of cases was virtually complete. However, the tendency of the physician to order a stool sample examination may be thought to vary. As discussed above we did see a partial overlap of clusters with high incidence of *Campylobacter *and diarrhea and we conclude from this that an increased tendency to analyze stool samples or increased general gastrointestinal morbidity in the area may have contributed to create the *Campylobacter *cluster we found.

6) Finally, a problem arises because we just know the addresses of the patients, not the places they were actually exposed. To the degree that these places are not identical, there's a non-differential misclassification at play in the dataset, which will work to blur any real associations. It therefore becomes important to obtain sufficient statistical power and a successful analysis will depend on having a fairly large dataset to work with. This will make it difficult to use our method for less prevalent diseases. By the use of relatively large cells, 5 km by 5 km, we also hoped to increase the chances that exposure would occur within the same geographical cell as the patient lived. One important sub-group of misclassified cases consists of those infected during foreign travel. We were able to address this problem directly by excluding cases of *Campylobacter *infections registered as infected abroad.

## Conclusion

Spatio-temporal datasets may contain important information on the distribution of the surveyed phenomena. In the present study, cases of *Campylobacter *and diarrhea for the Island of Funen for a period of nine years were analyzed. Because the exact residence is known for all inhabitants in Denmark, we were able to calculate precise incidences over space and time. Our results show a persistent cluster of *Campylobacter *features in the North-Western part of our study area, partly overlapping with a cluster of diarrhea features. These findings might indicate an environmental cause of campylobacteriosis in the area, and should serve as a starting point for further epidemiological studies.

## Competing interests

The authors declare that they have no competing interests.

## Authors' contributions

The authors jointly designed the study; MRJ and SE drafted the manuscript, MRJ programmed the Netlogo tool and produced the results, JS geo-coded and aggregated the data and developed the variance estimate. SE conceived of the study and MRJ defined the spatial statistics to use.

## Supplementary Material

Additional file 1 **Data-viewing possibilities of the Netlogo tool developed**. Data shown in Figure 7 are used as an example. Click here for file
